# Domain-General Mechanisms for Speech Segmentation: The Role of Duration Information in Language Learning

**DOI:** 10.1037/xhp0000325

**Published:** 2016-11-28

**Authors:** Rebecca L. A. Frost, Padraic Monaghan, Tomoko Tatsumi

**Affiliations:** 1 Department of Psychology, Lancaster University; 2 Department of Psychology, Lancaster University, and Department of Psychology of Language, Max Planck Institute for Psycholinguistics; 3 Department of Psychology, University of Liverpool

**Keywords:** iambic/trochaic law, language acquisition, speech segmentation, transitional probabilities, visual sequences

## Abstract

Speech segmentation is supported by multiple sources of information that may either inform language processing specifically, or serve learning more broadly. The Iambic/Trochaic Law (ITL), where increased duration indicates the end of a group and increased emphasis indicates the beginning of a group, has been proposed as a domain-general mechanism that also applies to language. However, language background has been suggested to modulate use of the ITL, meaning that these perceptual grouping preferences may instead be a consequence of language exposure. To distinguish between these accounts, we exposed native-English and native-Japanese listeners to sequences of speech (Experiment 1) and nonspeech stimuli (Experiment 2), and examined segmentation using a 2AFC task. Duration was manipulated over 3 conditions: sequences contained either an initial-item duration increase, or a final-item duration increase, or items of uniform duration. In Experiment 1, language background did not affect the use of duration as a cue for segmenting speech in a structured artificial language. In Experiment 2, the same results were found for grouping structured sequences of visual shapes. The results are consistent with proposals that duration information draws upon a domain-general mechanism that can apply to the special case of language acquisition.

## Public Significance Statement

This study shows that adults prefer to group both sequences of shapes and sequences of speech with the final item as the one that has the longest duration. This suggests that final-item duration increase is a helpful cue for perceptual grouping in multiple domains—not just language processing. By testing native speakers of languages that use duration differently, we also show that this grouping preference does not arise from language experience.

Identifying words from continuous speech is an enormously complex task, which is attested to by the imperfect accuracy of even very powerful automatic speech recognition systems (Hinton et al., 2012). Fortunately, words in speech contain a constellation of cues that learners can draw upon to assist in this task, including allophonic variation (Salverda, Dahan, & McQueen, 2003), phonotactic constraints on co-occurring segments (Hockema, 2006), transitional probabilities (Saffran, Aslin, & Newport, 1996), and prosodic information about stress and rhythm (Mattys, Jusczyk, Luce, & Morgan, 1999; Saffran, Newport, & Aslin, 1996; see Mattys, White, & Melhorn, 2005, for a review).

A number of the cues used for speech segmentation have been found to apply more broadly in perceptual learning, including information concerning prosodic stress, and variation in duration of both consonants and vowels (White, Mattys, Stefansdottir, & Jones, 2015). Drawing on an extensive body of work into rhythm perception and metrical phonology (Allen, 1975; Bolton, 1894; Fraisse, 1963, 1982; Woodrow, 1909, 1951), Hayes (1995) hypothesized that listeners preferred to group sounds with increased duration as sequence-final (iambic grouping), and preferred to group sequences varying in intensity or pitch with the emphasized element as sequence-initial (trochaic grouping)—termed the Iambic/Trochaic Law (ITL). The ITL was conceived as a domain-general mechanism, such that the same grouping preferences are applied to all stimuli, regardless of their type (speech or tones) or modality (visual or auditory).

A key question concerns how language learners come to apply these grouping preferences to help with identifying words from continuous speech. One possibility is that grouping preferences, such as those expressed in the ITL, are general-purpose mechanisms, as initially proposed by Hayes (1995). Under this view, the ITL is applied in order to acquire both linguistic and nonlinguistic stimuli, with speech signal processing being a special case of this general preference, used to assist language acquisition (Endress & Hauser, 2010; Tyler & Cutler, 2009). Alternatively, these grouping preferences may be *learned,* as a consequence of exposure to the speech signal, with knowledge of the usefulness of cues developing alongside acquisition of the language (Saffran et al., 1996). In line with the latter perspective, some researchers have proposed the ITL to be a consequence of learning the role that duration and intensity variation has with respect to the position of syllables in words (Kusumoto & Moreton, 1997), or the position of words in phrases (Iversen, Patel, & Ohgushi, 2008), and then applying this information learned from speech to other, nonlinguistic stimuli (Peña, Bion, & Nespor, 2011).

Evidence for the learning hypothesis—that grouping preferences are acquired as a consequence of exposure to language structure—has been derived from studies that determine variation in the extent to which different languages conform to the ITL, and matching this to the stimuli-grouping preferences for speakers of those languages. Although there are multiple experimental demonstrations suggesting that the preference for the prominent element to commence a sequence exists as a perceptual preference regardless of language background (Hay & Diehl, 2007; Hayes, 1995; Iversen et al., 2008; Peña et al., 2011; Tyler & Cutler, 2009, though see Trainor & Adams, 2000) the prevalence of the preference for duration increase to mark the end of a group appears to be more variable cross-linguistically.

There is substantial evidence that the ITL guides the grouping of auditory sequences of alternating items for listeners of English, French, and German (Bhatara, Boll-Avetisyan, Unger, Nazzi, & Höhle, 2013; Bhatara, Yeung, & Nazzi, 2015; Boll-Avetisyan, Bhatara, Unger, Nazzi, & Höhle, 2015; Hay & Diehl, 2007; Hayes, 1995; Woodrow, 1909). The ITL also applies to metrical feet containing more than two elements, such as anapests (weak-weak-strong) and dactyls (strong-weak-weak), though the experimental evidence that these cues shape grouping is weaker for anapests than for two element sequences (Bolton, 1894; Trainor & Adams, 2000; Woodrow, 1909; but see Saffran et al., 1996, for use of final syllable lengthening as a cue to grouping of trisyllabic stimuli).

However, iambic grouping preferences (i.e., grouping according to duration increases in the item-final position) have not been reliably observed for listeners of Japanese (Iversen et al., 2008; Kusumoto & Moreton, 1997; Yoshida et al., 2010) and Basque (Molnar, Lallier, & Carreiras, 2014), whereas for Zapotec (Crowhurst & Teodocio Olivares, 2014), the iambic preference was found to be dependent upon the extent to which intensity also varied in the speech sequences. Explanations for the presence or absence of these effects have been linked to the properties of the duration information contained within these languages. English and German speech tends to comprise words that have increased duration of final syllables compared with the syllables that precede them (Bhatara et al., 2013; Monaghan, White, & Merkx, 2013; Tyler & Cutler, 2009), with further increases in duration for syllables that occur before phrasal or utterance boundaries. French, though having a different variation in duration associated with word boundaries (Hay & Diehl, 2007), still has increased duration associated with phrasal boundaries, co-occurring with word-final syllables, attributable in part to function word content word alternation (Iversen et al., 2008). Thus, from these languages the duration of syllables within words corresponds to the ITL (Turk & Shattuck-Hufnagel, 2000), and participants’ tendencies to make groupings according to final-element duration increases could thus reflect either domain-general preferences or learning as a consequence of language structure.

In contrast to English, French, and German, Japanese has been argued to lack variation in duration of morae corresponding with word boundaries (Iversen et al., 2008; Yoshida et al., 2010). This is partly because Japanese has been claimed to have isochronous productions of morae (Bloch, 1950; Hockett, 1955; Ladefoged, 1975, 1993), and also because of differences in word order, with functors typically occurring before content words in English and after content words in Japanese (Baker, 2001). As functors tend to be reduced in speech, this could result in a lack of duration increase phrase-finally in Japanese (Iversen et al., 2008). Claims of isochrony in morae appear to be somewhat exaggerated, with substantial variation in mora durations evident in Japanese speech (Han, 1962; Jinbo, 1927/1980). Indeed, phrase-final morae are indeed likely to have increased duration compared with non-final morae (Arai & Greenberg, 1997; Beckman, 1982, 1992). In a corpus analysis, Kaiki and Sagisaki (1992) found that morae did have duration increase in the final position of a “breath group”, which often corresponded with a phrase boundary (see Warner & Arai, 2001, for review). However, it remains the case that Japanese content words are less likely to be preceded by (shorter) function words than in languages such as English or French (Monaghan, Christiansen, & Chater, 2007), reducing the alternation of short and long syllables, or morae, in speech.

Hence, it could be that word-final duration increase is reduced in Japanese compared with English, French, or German because of differences in word order. In this case, this difference in the degree, rather than the direction, of the effects, may be sufficient to drive use of duration increase to indicate word-final syllables in English, French and German, but not in Japanese. An alternative hypothesis to the domain-general account of the ITL is that the iambic preference is instead learned from experience with natural language. As the effect demonstrated in production data is smaller in Japanese, Japanese listeners may fail to derive a preference for final-element grouping according to duration—providing a possible explanation for the results of Iversen et al. (2008) and Yoshida et al. (2010) based on learning of duration variation.

However, the majority of previous studies testing the influence of variation in duration have used unstructured stimuli that comprise alternating pairs of tones or syllables, with equal transitional probabilities between each tone or syllable (Hay & Diehl, 2007; Iversen et al., 2008; Peña et al., 2011; Yoshida et al., 2010). A benefit of such a design is that it isolates the effect of duration for grouping. However, a fundamental disadvantage is that it does not enable an assessment of how cues are used to support and discover learning of structure within sequences. In this respect, these unstructured stimuli are atypical of natural language, which contains substantial structure in terms of different transitional probabilities and dependencies between syllables within words. Understanding the way that prosodic cues are used in coordination with statistical information is critical for determining how duration information operates relative to the task of speech processing (see, for instance, Crowhurst & Teodocio Olivares, 2014 for how the role of speech cues can change in interaction compared with when they are presented independently).

An important aspect of language structure in natural (Pelucchi, Hay, & Saffran, 2009) and artificial (e.g., Saffran et al., 1996) language learning studies is varying transitional probabilities between syllables in speech. In language, syllables that frequently co-occur together tend to be grouped together within a word, whereas pairs of syllables that do not reliably occur together are used to indicate word boundaries. Research using artificial language has shown that transitional probabilities are utilized for speech segmentation by learners of all ages (e.g., Frost & Monaghan, 2016; Saffran et al., 1996). Saffran et al. (1996) tested the extent to which learners processed final-syllable duration increase in stimuli that contained this feature of natural language structure. American-English listeners heard continuous streams of artificial speech, in which transitional probabilities between syllables were varied. The speech was composed of words which were triplets of syllables that always co-occurred (within word transitional probability = 1), and each word could be followed by one of three other words (between word transitional probability = 0.33). The role of syllable duration within words was assessed in three conditions; a control condition, where all syllables were identical in duration (all 278 ms), an initial syllable duration increase condition, and a final syllable increased duration condition, with the duration of lengthened syllables being increased by 100 ms. Following familiarization, learning was examined with a two-alternative forced-choice task containing word versus part-word comparisons. Findings indicated that learning was best when the final syllable was increased in duration, and marginally worse when the initial syllable was increased in duration, suggesting that duration information supported identification of words that were defined in terms of the transitional probabilities between syllables.

Thus, for English listeners, final syllable duration increase was shown to enhance processing of the statistical structure of speech. The aim of our study was to test whether the different operation of duration-related perceptual grouping observed previously for listeners of languages with varying realizations of phrase-final duration increase extended to segmentation of sequences that were defined in terms of different transitional statistics, which are more reminiscent of natural language structure.

In this study, we constructed two experiments designed to distinguish between the domain-general mechanism account and the learning account of the iambic grouping preference. In Experiment 1, we tested English and Japanese speakers’ ability to segment sequences of syllables when those sequences contained statistical structure relating to that of words in natural language. This first experiment attempted to replicate Saffran et al.’s (1996) observation that English-speaking participants use final-syllable duration increase to support segmentation of continuous speech that contains words defined by transitional probabilities. Experiment 1 also tested whether Japanese listeners receiving the same language structure as the English listeners would also make use of final-syllable duration increase, or whether no combination of iambic grouping cue and statistical structure would benefit this group. If the Japanese listeners can use final-syllable duration increase as a cue for speech segmentation, then this suggests that the use of the iambic grouping preference is not dependent upon language exposure, and is more likely a domain-general mechanism. If the Japanese listeners cannot use the final-syllable duration increase cue, then this instead shows that the iambic grouping preference is more likely to be dependent upon language experience, in line with Iversen et al.’s (2008) and Yoshida et al.’s (2010) explanation for their data. Intriguingly, Iversen et al. (2008) found that individual Japanese listeners reliably applied either a preference for long-short or short-long sequences for grouping, but with no overall tendency for either type. If there is also statistical information available in the speech, such that the sequences are more language-like, then it is possible that Japanese participants may more reliably apply a grouping principle to the sequences.

Thus, in Experiment 1, we explored the extent to which increasing duration of syllables in continuous speech can support the learning of words that are defined in terms of transitional probabilities. If the use of durational information for grouping arises as a consequence of language exposure, then we would expect a final-syllable duration increase to improve learning for English listeners but result in a small, or no, effect for Japanese listeners, similar to the results of Iversen et al. (2008) and Yoshida et al. (2010) for unstructured stimuli. If, however, the use of duration information for perceptual grouping is domain-general, and not dependent on exposure, then learning for English and Japanese listeners should be equivalent.

We know that transitional probability information can be relied upon for learning in a range of different modalities, including sequences of tones (Saffran, Johnson, Aslin, & Newport, 1999), and shapes (Fiser & Aslin, 2002). A strong test of the domain-generality of the ITL was provided by Peña et al. (2011), who assessed how duration and intensity variation affected processing of visual sequences. They presented Italian-speaking adults with unstructured sequences of 10 shapes, which were continuously repeated over 3 min. To test duration effects on perceptual grouping, events alternated in duration from 320 ms to 800 ms. At test, participants were presented with static pairs of shapes, and were asked whether they occurred consecutively during training. Results indicated that participants were better at recognizing short–long than long–short sequences (with short–long and long–short referring to presentation during training).

As Peña et al. (2011) noted, the results could be explained by cross-modal transfer from learning regularities in duration variation from natural language structure which is then applied to non-auditory sequences. However, such a view seems inconsistent with recent reviews of studies that indicate learning of particular statistical structures do not readily transfer across modalities (Frost, Armstrong, Siegelman, & Christiansen, 2015). Their results are therefore more likely to be consistent with a domain-general mechanism applying across stimulus types, such as in the original conception of the ITL (Hayes, 1995).

Peña et al.’s (2011) study used sequences with no structural information, to isolate the role of duration on grouping. Consequently, the interaction between statistical structure and additional grouping cues cannot be observed. Thus, in Experiment 2, we tested whether final-element duration increase assisted learning of visual sequences that were defined in terms of transitional probabilities, for both English and Japanese listeners. If language exposure determines the effect of duration increase for identifying sequence structure then we anticipate that English listeners will use final-element duration increase more than Japanese speakers. If the use of final-element duration increase is instead domain-general, then we expect both English and Japanese speakers to use the cue to a similar degree.

## Experiment 1: Use of Duration Information in Speech Segmentation

In this experiment, we replicated previous studies of native-English listeners’ use of a final-syllable duration increase cue for segmenting artificial speech, and extended this test to a group of native-Japanese listeners. If use of final syllable duration increase is language dependent then we expect a smaller effect for Japanese than English speakers. If the effect is domain general, and not learned from language exposure, then we expect similar use of the cue by both English and Japanese speakers.

### Method

#### Participants

From power analyses based on Saffran et al.’s (1996) study of duration cues, we determined that 12 participants per condition would result in power of .79 for finding a difference between any two conditions (effect size in Saffran et al.’s (1996) study resulted in Cohen’s *d* = 1.113 for the comparison between their final duration increase and no duration increase conditions). For the English group, 36 students from Lancaster University, 11 males and 25 females with a mean age of 20.69 years (*SD* = 4.06), volunteered to participate in the study for course credit. All participants reported English as their first language and reported no hearing or vision problems. For the Japanese group, 34 native Japanese listeners who were students and staff at the Tokyo University of Foreign Studies, 11 males and 23 females with a mean age of 22.69 years (*SD* = 4.655), volunteered to take part in the study, and received 700 yen for their participation. Data for a further two participants were collected, but were removed from the analysis because they were outliers in terms of age (ages 61 and 59). The Japanese listeners all had some knowledge of a second language, required as part of the high-school curriculum, however, we did not collect information on level of proficiency in other languages for the participants. Although experience with a second language is likely to have a very limited effect on processing here (see Boll-Avetisyan et al., 2015, and Molnar et al., 2014), we return to the issue of influence of second language learning on performance in the General Discussion.

#### Materials

We constructed an artificial language from six consonants (/b, d, g, k, p, t/), each used twice, and four vowels (in English:/æ, i, ɔ, u/, in Japanese:/a, i, o, ɯ/), each used three times, which were combined to produce 12 distinct CV syllables. The consonants were selected as those that were attested in both English and Japanese speech, and plosives were selected because these had a distinctive onset enabling duration of the syllable or mora to be processed by the listener. Vowels were selected to ensure distinctiveness in the productions of the speech synthesizer by varying both height and position of the vowels. The syllables were then concatenated to create four trisyllabic words (e.g., *bogada, dugibu, kitapo, pikotu*), which were compiled pseudorandomly into a speech stream, with the restriction that no word was directly repeated. Within words, transitional probabilities between syllables were 1, and between words transitional probabilities were .33. Four different versions of the language were generated and counterbalanced across participants, to ensure that no biases for particular sequences influenced participants’ performance (Onnis, Monaghan, Richmond, & Chater, 2005). These versions had different combinations of consonants and vowels within syllables or mora, and different combinations of syllables/mora comprising the four trisyllabic words. We ensured that words in the experimental languages were not preexisting words in English or Japanese.

For the English listeners, speech was synthesized using the Festival speech synthesizer (Black, Taylor, & Caley, 1990), using the kal British English diphone database. For the Japanese listeners, speech was synthesized with MBROLA (Dutoit, 1997) using the jp1 diphone database. For the synthesized speech, the diphone database permitted allophonic variation to be present, to result in more naturalistic speech. Duration increases were implemented in the training speech only. Duration of syllables was 233 ms, and 333 ms for increased duration syllables, similar to the speech used by Saffran et al. (1996). Duration increase was implemented by increasing the vowel duration during synthesis by 100 ms. Speech was produced in a monotone with mean F0 of 120 Hz. Continuous speech was produced from streams of 150 words, in one of three conditions: initial-syllable duration increase (ISI), where the first syllable in every word was increased in duration; no duration increase (NI), where every syllable had equal duration; and final-syllable duration increase (FSI), where the third syllable in each word was extended in duration. There were no pauses between any of the syllables in the speech. Training time was 120 s for the initial and final syllable duration increase conditions, and 105 s for the no duration increase condition, and speech streams were edited to fade in and out for the first and last 5 s.

Critically, for the test materials the four word stimuli were synthesized with equal syllable duration (all 233 ms), regardless of the training condition. This was so that we could determine how the duration information was used to detect the structure, similar to previous speech segmentation studies applying syllable duration variation (Saffran et al., 1996). Including the duration variation during testing would have meant that responses could be driven entirely by preferences for particular sequences regardless of the structure of the language. Sixteen part-words were also generated with equal syllable durations of 233 ms. Part-words occurred in the training speech but straddled word boundaries, comprising the last syllable of one word and the first two syllables of another word (so, for words of the form ABC, part-words would be of the form CAB), or the last two syllables of one word and the first syllable of another (BCA). Table 1 shows the relationship between the duration cue’s position during training for the word and part-word test items.[Table-anchor tbl1]

#### Procedure

Participants within each language group were randomly assigned to one of three duration conditions. All instructions were presented in the listeners’ native language. Instructions given in Japanese were based on direct translations of instructions given to the English listeners (performed by a native speaker of Japanese) to ensure precise comparability. Participants were instructed as follows: “Listen to the speech and try to determine the structure of the language.” After exposure to the speech, participants completed a forced-choice test containing 16 trials. For each trial, participants listened to a word and a part-word, separated by a 1-s pause and were instructed to “Select which sequence best matches the language you have just heard,” giving a key-press response of “1” for the first or “2” for the second sequence. Order of words and part-words within pairs was counterbalanced. In the test, all words occurred 4 times, and each part-word occurred once. Training and testing was then repeated, to examine effects of repetition of words, and to assess whether any additional learning took place over the course of the test phase. If performance improved at the second test then this could indicate that exposure to words and part-words affected performance on the task. If there was no effect then this means that the test itself was not affecting participants’ processing of individual stimuli. The experiment lasted for approximately 8 min. All participants were tested individually in an isolated booth, and received training and test items through closed-cup headphones. Participants listened to the speech at a volume that they found comfortable.

### Results

To test the overall effect of duration on performance, we conducted a repeated-measures ANOVA on accuracy scores (proportion of selections of words over part-words), with language group (English or Japanese) and cue condition (ISI, NI, or FSI) as between subjects factors, and test time (first and second test) as a within subjects factor.

There was no significant effect of test time (Test 1: *M* = .652, *SE* = .025; Test 2: *M* = .678, *SE* = .026, *F*(1, 64) = 2.184, *p* = .144, η_p_^2^ = .033). Interactions involving test time were not significant and are not further reported (all *p* > .05). This indicated that no learning took place during the testing.

**Figure 1 fig1:**
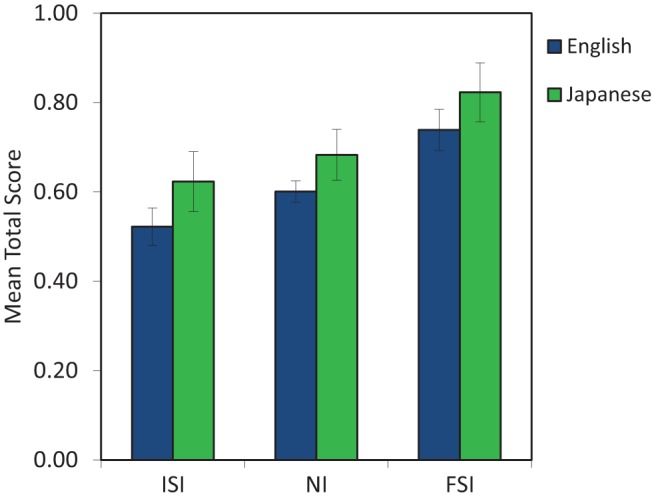
Mean word-identification score for participants in each cue condition, given for English and Japanese listeners. Error bars show ±1 *SEM*. See the online article for the color version of this figure.

There was a significant effect of cue condition, *F*(2, 64) = 8.323, *p* = .001, η_p_^2^ = .206, and a linear contrast respecting the predicted order of cues (ISI, NI, FSI) was highly significant, *F*(2, 64) = 8.323, *p* = .001, η_p_^2^ = .206 (see Figure 1). Dunnett’s post hoc *t* tests conducted to respect the linear contrast order revealed that the FSI condition (*M* = .781, *SE* = .040) resulted in more accurate word identification from the speech than the NI condition (*M* = .638, *SE* = .030), *p* = .009, and the ISI condition (*M* = .573, *SE* = .040), *p* < .001. The NI and ISI conditions did not differ significantly, *p* = .199.[Fig-anchor fig1]

There was a significant effect of language group, *F*(1, 64) = 4.321, *p* = .042, η_p_^2^ = .063, with Japanese listeners (*M* = .712, *SE* = .039) more accurate than English listeners (*M* = .621, *SE* = .026) at identifying words from speech. There was no significant interaction between language group and cue condition, *F*(2, 64) = .019, *p* = .981.

### Discussion

For English listeners, the results replicated previous studies demonstrating the benefit of final-syllable duration increase for identifying word boundaries in speech, where word boundaries are defined in terms of transitional probabilities between syllables (Saffran et al., 1996). The linear effect of cue condition showed that increasing the duration of the initial syllable had a slight detrimental effect on identifying word boundaries compared with the other conditions (though this was not significantly different than chance), whereas increasing the duration of the final syllable meant that such boundaries were isolated more accurately compared with when no duration cue was present in the speech.

Interestingly, the results for Japanese listeners showed a very similar pattern. As with the English listeners, increasing the duration of the final syllable improved accuracy for word identification, where word boundaries were defined by the same transitional probabilities present in the speech heard by native-English listeners. Together with the fact that there were no interactions concerning native language and cue condition, this finding indicates that both language groups were using duration information in the same way to segment speech, contrary to findings of previous comparisons of English and Japanese listeners for unstructured sequences (Iversen et al., 2008; Kusumoto & Moreton, 1997; Yoshida et al., 2010).

A key difference between our study and previous comparisons of English and Japanese listeners is the statistical structure of the stimuli. For Iversen et al. (2008), Kusumoto and Moreton (1997), and Yoshida et al. (2010), participants listened to pairs of tones or syllables, with no transitional probability information. In our study, transitional probabilities were varied, such that there was a statistical structure to the speech to be discovered by participants. This could have been sufficient to guide the sporadic use of either an iambic or a trochaic grouping preference seen for Iversen et al.’s (2008) Japanese listeners toward a preference for grouping based on duration increase in the final element (to reflect structure). For unstructured sequences, such as those in Iversen et al.’s (2008) study, it is just not possible to integrate the prosodic information with the statistical information present in the speech potentially obscuring use of a domain-general iambic preference for detecting structure.

Importantly, the presence of structure in the speech did not lead English or Japanese listeners to prioritize a grouping preference for final duration increase regardless of the informational structure of the speech. Participants hearing speech with increased duration of the initial syllable demonstrated a non-significant deficit in word identification compared with those in the no duration increase condition, but performance was not lower than chance, meaning that the duration cue did not entirely override the statistical information; rather, both statistical and prosodic cues were used in combination. Thus, duration-related perceptual grouping is used in conjunction with, and not as a consequence of, the statistical structure of the speech.

Another important methodological distinction between our study and previous cross-linguistic comparisons of use of duration information in speech was the fact that in our study no durational information was present in the stimuli during testing. Rather, we tested learning as a consequence of using duration information, instead of testing the immediate influence of the duration cue on sequence perception. This enables us to determine how duration can be utilized to support learning of sequential structure, and avoids effects of perceptual capture during testing.

Our results are consistent with the idea that the use of duration variation as a cue is independent of language exposure; both English and Japanese listeners used final syllable duration increase to a similar degree, regardless of the magnitude of this effect in their background language. The results are thus indicative of duration information being available as a domain general cue. The next experiment tested whether duration variation exerted a similar effect for grouping visual sequences of shapes for English and Japanese listeners, to determine whether the effect of duration is specific to language stimuli, or is generalizable across modalities. If the use of duration information is language specific (as would be expected if the iambic preference is learned from language structure), then we would not find an effect of duration on grouping of structured sequences of shapes. However, if the preference is modality independent (as would be expected from a domain-general mechanism that is not learned as a consequence of language exposure), then the results should mirror those of the language stimuli in Experiment 1.

## Experiment 2: Use of Duration Information in Grouping Visual Sequences

### Method

#### Participants

For the English listeners, 36 students from Lancaster University, 8 males and 28 females with a mean age of 19.89 years (*SD* = 2.48) volunteered to participate in the study for course credit. All participants were native-English speaking, and reported no auditory or visual problems. For the Japanese listeners, 36 native-Japanese speaking participants who were students and staff at the Tokyo University of Foreign Studies, 13 males and 23 females with a mean age of 22.44 years (*SD* = 4.339), volunteered to take part in this study, and received 700 yen for their participation. As in Experiment 1, the Japanese listeners had some knowledge of a second language, required as part of the high-school curriculum, however we did not collect information on level of proficiency in other languages. We return to the issue of influence of a second language on performance in the General Discussion (but see Boll-Avetisyan et al. (2015) and Molnar et al., (2014) for accounts suggesting that effects of this nature are likely to be minimal). No participants had previously taken part in Experiment 1.

#### Materials

**Figure 2 fig2:**
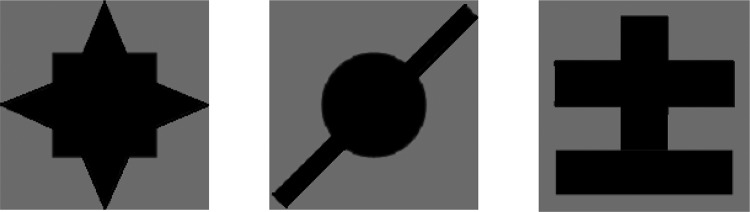
Examples of shape stimuli used in Experiment 2.

The materials were identical for both language groups, and were constructed to match the structural properties of the language used in Experiment 1, with each syllable being replaced by a shape. We selected 12 geometric shapes printed in black on a gray background, taken from Fiser and Aslin (2002), each 170 × 170 pixels in size. Figure 2 shows some example stimuli used in the study. Shapes were concatenated into four triplets, with four different random arrangements counterbalanced across participants to ensure that no biases in terms of sequence preferences adversely affected the results. For the shapes, duration increase was implemented by displaying shapes on screen for 100 ms longer than the other shapes. Shapes with standard duration appeared at the center of a computer screen for 225 ms, and shapes with increased duration appeared for 325 ms. We wanted to ensure that the durational increases implemented for shapes were similar to those implemented for the speech stimuli, and while Peña et al. (2011) varied length of shape stimuli from 320 ms to 800 ms, such large duration differences could have resulted in divergence of processing from the speech stimuli. Though the overall duration is controlled between the speech and shape stimuli, it is important to note that increased duration of the speech resulted in a change across the vowel, which is not a stable state, whereas the presentation of shapes was stable for their duration. Such a difference enables us to test the extent to which duration is durable as a cue for grouping, over different modalities, and over dynamic versus static stimuli.[Fig-anchor fig2]

As with the speech, a familiarization sequence was created for each condition, comprising 150 shape triplets, with no shape triplet immediately repeated. Transitional probabilities between shapes within a triplet (transitional probability = 1) and between triplets (transitional probability = .33) were identical to those in Experiment 1. A blank screen occurred for 225ms between the presentation of every shape, as pilot studies demonstrated that without this the stimuli were uncomfortable to view. This meant that the stimuli were somewhat different than the speech streams in terms of continuity, however, again, this enables us to provide a stronger test of the robustness of the use of duration information for grouping stimuli across modalities. Streams in the initial- and final-item duration increase conditions lasted for 218 s, and streams in the no duration increase conditions lasted for 203 s.

For testing, we generated sequences that corresponded to words and part-words with the same structure as in Experiment 1: triplets that reliably occurred together during training were the “words,” and triplets that crossed boundaries between triplets were “part-words.” As with the speech, during testing all shapes were presented for the same duration (225 ms, with 225 ms blank screen interval): no duration cue was present at this stage. Pairs of sequences were separated by a 1000-ms pause.

#### Procedure

Participants were instructed as follows: “You will see sequences of shapes, and your task is to try to determine their structure.” Participants were assigned to a final-shape duration increase, a no duration increase, or an initial shape duration increase condition, and viewed the corresponding training sequences on a computer screen. At test, participants were asked: “Select which of two sequences best fits the structure of the sequences you just saw.” They then viewed the 16 forced-choice items, responding with a keyboard press as in Experiment 1. Training and testing was then repeated. All instructions were presented in the participants’ native language.

### Results and Discussion

A repeated-measures ANOVA was performed on the data (proportion of correct responses), with cue condition (ISI, NI, FSI) and language group (English, Japanese) as between subjects factors, and test time (Test 1, Test 2) as a within subjects factor.

There was a significant effect of test time (Time 1: *M* = .597, *SE* = .016; Time 2: *M* = .705, *SE* = .021, *F*(1, 66) = 22.754, *p* < .001, η_p_^2^ = .256). This may have been attributable to the effect of learning during the test, as “words” occurred more frequently than “part-words.” However, critically, all interactions involving test time were not significant and are not further reported (all *p* > .05), thus any learning during test did not affect performance in any of the duration conditions or language groups differentially.

There was no significant effect of language group, *F* < 1, indicating that English and Japanese listeners performed to a similar degree across the three conditions (ISI: English *M* = .595, *SE* = .019, Japanese *M* = .625, *SE* = .031; NI: English *M* = .635, *SE* = .042, Japanese *M* = .634, *SE* = .034; FSI: English *M* = .713, *SE* .044, Japanese *M* = .702, *SE* = .041).

**Figure 3 fig3:**
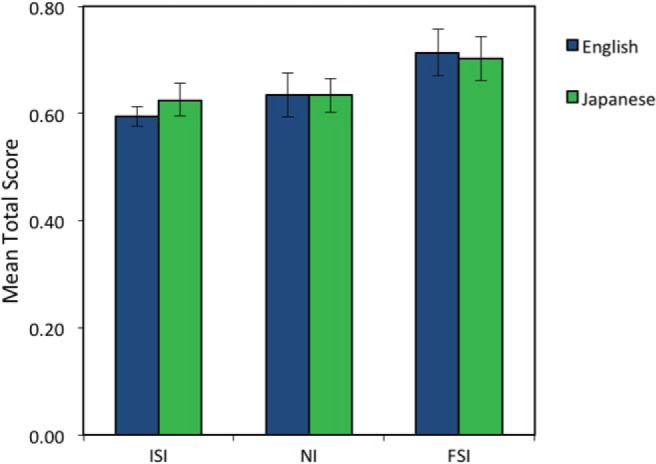
Mean shape-sequence identification score for participants in each cue condition, given for English and Japanese listeners. Error bars show ±1 *SEM*. See the online article for the color version of this figure.

There was a significant effect of cue condition, *F*(2, 66) = 3.820, *p* = .027, η_p_^2^ = .104, and a linear contrast respecting the predicted order of cues (ISI, NI, FSI) was significant, *F*(2, 66) = 3.820, *p* = .027, η_p_^2^ = .104 (see Figure 3). Dunnett’s post hoc *t* tests conducted to respect the hypothesized linear contrast order revealed that participants in the FSI condition (*M* = .708, *SE* = .029) were more accurate than those in the NI condition (*M* = .635, *SE* = .026), *p* = .047, and the ISI condition (*M* = .610, *SE* = .018), *p* = .009. The NI and ISI conditions did not differ significantly, *p* = .379.[Fig-anchor fig3]

Critically, there was no significant interaction between language group and cue condition, *F*(2, 66) = .209, *p* = .812, indicating that language background did not differentially affect use of duration information for learning the structure of visual sequences. The other interactions were not significant (all *p* > .05).

The results were very similar to those of the language stimuli in Experiment 1. The English listeners demonstrated the same benefit of element-final duration increase for grouping the statistically defined visual sequences. These results are consistent with previous studies of unstructured visual sequence processing, as shown by Peña et al. (2011). In Peña et al.’s (2011) study, Italian participants, whose native language contains final-syllable duration increase (Tyler & Cutler, 2009), demonstrated an iambic grouping preference for visual sequences varying in duration. Thus, our results corroborate these earlier findings, and demonstrate that they are generalizable to visual shape sequences with more complex statistical structure.

The results for the Japanese participants are, however, more difficult to reconcile with the hypothesis that grouping according to final element duration is a consequence of exposure to this structure in natural language. The remarkably similar use of final-element duration increase across modalities, and across listeners with different language backgrounds, suggests that the iambic preference operates independently of language exposure, and can be used to support statistical structure of sequences by participants with differing exposure to duration variation in their language experience.

## General Discussion

The experiments in this study tested two influential theories for how the iambic grouping preference might influence listeners’ perception of sequences. The first theory claims that the iambic preference for perceptual grouping is domain-general, and is applied to language as just one of several types of stimuli (Hayes, 1995; Tyler & Cutler, 2009). The alternative theory contends that the iambic preference is acquired as a consequence of language exposure (Iversen et al., 2008; Yoshida et al., 2010). Experiment 1 tested the extent to which variation in language exposure resulted in variation in use of duration information for grouping structured speech stimuli. We tested native listeners of English and Japanese, who are hypothesized to vary in terms of the size and reliability of the effect of final syllable or mora duration for perceptual grouping in their background language (Iversen et al., 2008; Yoshida et al., 2010). Our results demonstrated that the properties of the listeners’ native language did not affect application of final syllable, or mora, duration in supporting speech segmentation.

Previous studies testing listeners of languages with final-syllable duration increase have shown that for syllables, tones, and shapes, the use of the duration variation for grouping sequences is consistent with the way in which durational cues operate in natural language (Hay & Diehl, 2007; Peña et al., 2011). We replicated these results for both speech and visual sequences of shapes for English listeners, whose prior language exposure contains final-syllable duration increase as a useful cue for indicating word boundaries (Cutler & Carter, 1987; Monaghan et al., 2013; Saffran et al., 1996; Tyler & Cutler, 2009). We found that final-syllable duration increase improved segmentation of speech into words, compared with speech with no duration increase, or speech with a similarly informative cue of initial-syllable duration increase.

Experiment 1 also enabled us to examine whether previous demonstrations of Japanese listeners’ absence of reliable grouping based on final-mora duration increase (Iversen et al., 2008) could be explained by the lack of structure within the sequences. In this respect, unlike in prior research, our studies include a critical sensitivity to the way in which duration information is used to support learning for structured sequences—as is required for speech segmentation in natural language.

Experiment 2 tested whether duration information was able to support learning of sequential structure in another modality. If this was the case, then it increases the likelihood that the use of durational information for perceptual grouping is attributable to a domain general processing mechanism. We found that English listeners were able to use a final element duration increase to support learning of visual sequences defined in terms of transitional probabilities. These results are consistent with a domain general mechanism applying to sequence processing across a range of modalities (Hayes, 1995). Thus, it is possible that the same principle can be applied (depending on it’s effectiveness in indicating statistical structure) to speech processing, either for speech segmentation (Saffran et al., 1996), for determining phrasal structure (Hay & Diehl, 2007), or for detection of nonlinguistic sequence structures.

The results of Experiment 1 and Experiment 2 for the Japanese listeners are difficult to reconcile with a view of language-dependent learning as the source of the use of duration variation to indicate grouping (Iversen et al., 2008; Yoshida et al., 2010). Japanese listeners are likely to experience a smaller effect of mora duration increase in word-final position than English listeners, yet we found that their use of final element duration increase for segmentation was at least as reliable as for the English listeners. Our preferred explanation is consistent with the conventional view of the ITL; that final-syllable duration increase promotes segmentation because it aligns with general purpose processing mechanisms, that form sequence boundaries after duration increase.

Yet, this domain-general account is inconsistent with previous findings regarding Japanese listeners’ disregard of final element duration increase for grouping tonal and syllable sequences. Yoshida et al. (2010) discovered that 7- to 8-month-old English infants were sensitive to final-element duration increase in pairs of unstructured tones, whereas 5- to 6-month-old English infants and 5- to 6- and 7- to 8-month-old Japanese infants showed no such preference—seemingly suggesting that preferences emerge as a consequence of linguistic experience. Similarly, Bion, Benavides-Varela, and Nespor (2011) found that final-syllable duration increase had a positive effect on memory of speech sequences for adults, but no effect was found for 7-month-old infants. These results remain somewhat puzzling, and stand in contrast to developmental observations of use of duration cues in other nonlinguistic stimuli. For instance, for musical stimuli, grouping according to final-element duration increase has been found in children as young as 4.5 months (Jusczyk & Krumhansl, 1993; Krumhansl & Jusczyk, 1990). The developmental results of Yoshida et al.’s (2010) study may instead be attributable to cues becoming available only when the child’s cognitive capacity is able to integrate multiple cues.

This discrepancy between our results and previous studies with Japanese listeners could also be due to the task requirements for the participants. The lack of structure in the sequences used by previous studies with Japanese listeners may mean that use of the duration cue alone is insufficient to drive behavior in a consistent direction (note that Iversen et al., 2008 found consistency of responding within participants but not across participants). However, when the sequences also contain structure to be discovered by the listener, the duration cue *can* be applied. We suggest that it may be the interaction of information sources that results in consistent use of duration for perceptual grouping. This is not to say that the iambic preference only applies in special cases. Rather, most sequences in natural environments contain structural information (Tyler & Cutler, 2009; Zacks, Kumar, Abrams, & Mehta, 2009), and so decoupling this structure from the cues that support its processing and discovery results in a less natural test of the use of mechanisms potentially designed to support structure learning. Further investigations of duration information used by young Japanese and English infants with structured sequences will provide a resolution to these questions.

Another potential contribution to performance in the Japanese listeners in our study is that they were all degree-level students or graduates, and will have had some English language experience as a compulsory part of their high school curriculum. However, the influence of second language on the iambic grouping preference has been shown to be very limited. Boll-Avetisyan et al. (2015) showed that French listeners learning German as a second language demonstrated an influence of second language on the trochaic preference for syllable sequences varying in intensity, but there was no evidence that this affected grouping of syllables varying in duration. Furthermore, Molnar et al. (2014) showed that bilingual infants exposed to Basque and Spanish showed modulation of the iambic effect for duration differences in tone sequences depending on the infants’ dominant language. Thus, the effect of interference from a second language (even for bilingual listeners) appears to be minimal.

In addition to incorporating structure into the sequences, there are two other critical differences in the tasks conducted with our participants and those of previous studies. First, our use of triplets of syllables (or shapes), rather than pairs, may have affected participants’ sequence processing as effects demonstrated using anapaests may be weaker than those seen in studies that use iambic stimuli (Trainor & Adams, 2000). Second, the duration cue was only implemented in the training stream, meaning that after listening to the speech stream that contained duration variations, participants were tested with stimuli where all syllables (or shapes) had equal duration. This meant that the preference for final-element lengthening could not be immediately driving task performance, but rather exerted its effect indirectly during training, in establishing which were the likely sequences in the speech or visual shape groupings. In previous studies it is feasible that duration of an element could overshadow the statistical structure of sequences once they have been learned. It is, for instance, difficult to ignore prosodic information even for learned words: Misplacement of stress can impair word recognition profoundly (Slowiaczek, 1990), and irregular stress patterns can slow lexical access (Arciuli & Cupples, 2006). In this respect, our study is a purer test of the way that the duration-related grouping preferences can be used to discover sequence structure, rather than assessing immediate decisions based on the cue’s presence during testing.

In Experiment 2, we extended studies of durational effects in auditory stimuli to also test these effects in structured sequences of shapes. When the same statistical structure was incorporated into visual sequences of shapes, participants selected sequences that had appeared in training with final-element duration increase as the best candidates for items that were consistent with the familiarized structure. There are multiple reasons to doubt that the preference for final element duration increase evident for nonspeech stimuli is a transfer effect from natural language exposure. Conway and Christiansen (2006) and Frost et al. (2015) have shown that such cross-modal transfer is extremely difficult to achieve, at least in laboratory settings. In a comprehensive review, Frost et al. (2015) claim that there is no evidence for cross-modal transfer of statistical learning between visual, auditory, and tactile inputs, and suggest this is evidence for modality-specific learning. Thus, learning within one modality cannot readily be applied to processing structure in another modality.

In the case of our results then, the same preference for final-element duration increase in supporting statistical structure in speech and visual sequences is more economically explained by operation of a domain-general learning mechanism that exerts an effect in the same way on different modalities of stimuli. The alternative is to propose that two different learning biases, with the same effect, apply to distinct modalities. Distinguishing between these accounts may be difficult to accomplish, and so resorting to Occam’s razor, in selecting one bias over two biases, may be the most appropriate approach (see Saffran, 2002, for a similar argument).

Final-element duration increase occurs in a variety of modalities other than language, such as music (Palmer, 1997), and communications of birds and insects (Lindblom, 1978), and has been described in terms of a general law that longer durations are perceived as sequence final events (Hay & Diehl, 2007; Hayes, 1995; Woodrow, 1909). Such duration increase has been viewed as a consequence of production constraints (Lindblom, 1978), and our studies demonstrate that it is also functional for learning. We suggest that sensitivity to such duration variation for detecting structure is likely to be a consequence of general purpose learning mechanisms applying to language learning, with these mechanisms becoming adaptively encoded in the organism due to their stability within the environment. Such an adaptation can then speed up the detection and acquisition of informative structure from the environment.

Similarly, in the visual modality, human action and narrative events tend to be segmented at the onset of bursts of activity (Zacks et al., 2009), meaning visual sequences are more likely to conclude with situations that are present for longer durations than sequence onsets, with sequence onsets characterized by unpredictability (i.e., low transitional probabilities). Thus, encoding duration of visual scenes as a cue could also be beneficial for information processing of visual events in the environment. We suggest that, over time, this domain general production constraint could result in adaptation of a cognitive mechanism that is able to identify the realization of this constraint in structured sequences (Christiansen & Chater, 2008). For language, when the sequential information becomes exquisitely complex, the coopting of domain general processing mechanisms becomes more important than ever for supporting the discovery and processing of structure.

## Figures and Tables

**Table 1 tbl1:** Position of the Lengthened Syllable During Training for the Word and Part-Word Test Stimuli According to Cue Condition

Cue condition	Lengthened syllable (in bold)		
	Word	Part-words	
ISI	**A**BC	BC**A**	C**A**B
NI	ABC	BCA	CAB
FSI	AB**C**	B**C**A	**C**AB
*Note.* ISI = initial-syllable duration increase; NI = no duration increase; FSI = final-syllable duration increase
